# Microhematuria Enhances the Risks of Relapse and Renal Progression in Primary Membranous Nephropathy

**DOI:** 10.3389/fmed.2021.704830

**Published:** 2021-12-09

**Authors:** Peng He, Xiaoyong Yu, Yang Zha, Jing Liu, Hanmin Wang, Chen Huang, Shiren Sun, Lijie He

**Affiliations:** ^1^Department of Nephrology, Xijing Hospital, The Fourth Military Medical University, Xi'an, China; ^2^Department of Nephrology, Shaanxi Traditional Chinese Medicine Hospital, Xi'an, China

**Keywords:** primary membranous nephropathy, microscopic hematuria, relapse, kidney disease progression, remission

## Abstract

**Objective:** To determine whether there is an association between microhematuria and relapse or kidney disease progression in patients with primary membranous nephropathy (PMN).

**Methods:** A cohort of 639 patients with biopsy-proven PMN from two centers was followed for a median of 40 months. The exposures were initial hematuria, time-averaged hematuria, and cumulative duration of hematuria. The outcomes were relapse and renal progression, which were defined by a 40% reduction in renal function or end-stage renal disease. Cox proportional hazards regression and competing risk analyses were performed to yield hazard ratios (HRs) and subdistribution hazard ratios (sHRs) with 95% confidence intervals (CIs). Sensitivity and interaction analyses were also performed.

**Results:** After adjusting for confounders, a higher level of initial hematuria was associated with a 1.43 (95% CI, 1.15–1.78) greater hazard of relapse. Worsening hematuria remarkably increased the risk of short-term relapse (HR, 4.64; 3.29–6.54). Time-averaged hematuria (sHR, 1.35; 1.12–1.63) and cumulative duration of hematuria (sHR, 1.17; 1.02–1.34) were independent predictors of renal progression. Hematuria remission was related to a reduced risk of renal progression over time in patients with positive microhematuria (sHR, 0.63; 0.41–0.96).

**Conclusions:** A higher level of initial hematuria was a remarkable predictor of relapse in patients with PMN, and the magnitude and persistence of microhematuria were independently associated with kidney disease progression.

## Introduction

Glomerulonephritis (GN) may be caused by problems with the body's immune system. Damage to the glomeruli causes blood and protein loss in the urine. Microhematuria, accompanied by variable amounts of proteinuria, is one of the most common clinical manifestations of GN, e.g., IgA nephropathy (IgAN), ANCA-associated vasculitis (AAV), and lupus nephritis (1–3). The significance of hematuria in the natural history of renal disease is not clear (4–6). Some experts regard persistent hematuria as a hallmark of ongoing disease activity, but other experts posit that stable hematuria is a benign lesion resulting from prior kidney damage (5, 6). The role of hematuria in the development of glomerular diseases received unprecedented attention recently. Several studies showed that persistent hematuria was independently associated with kidney disease progression among patients with IgAN (7–9). Another study suggested that the presence of persistent hematuria, but not proteinuria, was a significant predictor of renal relapse in patients with AAV and kidney involvement (10).

Primary membranous nephropathy (PMN) is a kidney-specific, autoimmune glomerular disease, and it is one of the most common causes of nephrotic syndrome in non-diabetic adults worldwide (11–14). Approximately 80% of patients present with nephrotic-range proteinuria (≥3.5 g/d), and the remaining 20% have subnephrotic proteinuria (12–17). Renal function is normal at presentation in more than 90% of patients (12, 15–18). The most alarming long-term outcome of PMN is progressive loss of renal function, which occurs in ~60% of untreated patients, with 35% eventually reaching end-stage renal disease (ESRD) within 10 years (12, 13, 16, 17, 19, 20). The long-term renal survival of patients with persistent non-nephrotic proteinuria is >80–90% at 10 years (21). Other established predictors include age, male sex, decreased estimated glomerular filtration rate (eGFR) on presentation, persistent elevation of phospholipase A_2_ receptor antibody (anti-PLA_2_R) levels after therapy, and C3 staining in the biopsy sample (12, 13, 15–17).

Microhematuria is not uncommon, and appears in ~50–60% of patients with PMN (11–13, 15, 18). However, few studies systematically analyzed the prognostic relevance of microhematuria over time in PMN. The effects of microhematuria on disease activity and renal survival are not clear. In this study, we present a two-center longitudinal cohort of participants with biopsy-proven PMN. The present study (1) examined whether initial hematuria was an independent predictor for relapse of nephrotic range proteinuria and (2) investigated the magnitude and persistence of microhematuria over time in relation to kidney disease progression in patients with PMN.

## Methods

### Study Population

From 1 October 2015 to 30 June 2019, patients with biopsy-proven PMN from two centers in Xi'an, China, (the nephrology departments of Xijing Hospital [XH] and Shaanxi Province Hospital of Traditional Chinese Medicine [SPHTCM]) participated in this retrospective cohort. Other inclusion criteria were (1) a baseline (established at the time of renal biopsy) eGFR >15 ml/min/1.73 m^2^, calculated according to the Chronic Kidney Disease Epidemiology Collaboration equation (22), (2) at least 18 months of follow-up, and (3) sufficient information on treatments and laboratory parameters for investigation. Patients with secondary membranous nephropathy, atypical membranous nephropathy, or other concomitant glomerular diseases were excluded. The Ethics Committee of Xijing Hospital approved the study.

### Data Collection

Each participant attended regular visits at intervals of 3–12 months. Urine sediment and 24-h proteinuria excretion were tested at each visit and recorded prospectively. Skilled clinical examiners performed microscopic analyses of urine sediments. Each urine sample was strictly analyzed within 2 h of collection and reported as red blood cells (RBCs)/high-power field (HPF). We only recorded the results of urine sendiments, mainly of glomerular derived RBCs (dysmorphic RBCs > 70%). Since our study was focused on the microhematuria, the results of microscopic analyses during gross hematuria episodes were excluded. Other clinical data, including age, sex, body mass index, blood pressure, serum creatine, serum albumin, and use of renin-angiotensin-aldosterone system (RAAS) blockades and immunosuppressive (IS) agents during the first year, were systematically recorded. The follow-up data were last updated on 31 December 2020.

### Definitions and Outcomes

The follow-up time referred to the interval between kidney biopsy and the last outpatient visit, death, or ESRD, whichever occurred first. ESRD was defined as eGFR <15 ml/min/1.73 m^2^ or chronic dialysis. Hypertension was defined as systolic blood pressure ≥140 mmHg, diastolic blood pressure ≥90 mmHg, or the use of antihypertension drugs.

For PMN, nephrotic syndrome referred to proteinuria ≥3.5 g/d and serum albumin <3 g/dL. Complete remission (CR) was defined as proteinuria <0.3 g/d with serum albumin ≥3.5 g/dL and stable kidney function. A stable kidney function was defined as an eGFR that remained unchanged or declined by <40%. Partial remission (PR) was proteinuria >0.3 but <3.5 g/d plus a ≥50% reduction from baseline level, with serum albumin ≥3.5 g/dL and stable kidney function. No remission (NR) was defined as proteinuria ≥3.5 g/d, a <50% decline in proteinuria, serum albumin <3.5 g/dL, or a ≥40% decline in the eGFR prior to achieving proteinuria reduction. Relapse was defined as a reappearance of proteinuria ≥3.5 g/d after PR or CR.

The variables of interest were initial hematuria and the magnitude and persistence of microhematuria over time. Due to the high variability of microscopic assessment at a single time point, we treated the average hematuria of the first 6 months during follow-up as the initial hematuria. An initial hematuria of >5 RBCs/HPF was defined as initial persistent hematuria. The magnitude of microhematuria was expressed using time-averaged hematuria (TA-H). According to previously reported methods (8, 9), TA-H was the mean of the average hematuria, which was calculated for every 6-month block for each person. Time-averaged proteinuria (TA-P) was calculated in the same manner. The persistence of microhematuria was expressed using the cumulative duration of hematuria (CD-H), which was the sum of the number of months with a microhematuria count >3 RBCs/HPF. Persistent hematuria was defined by TA-H >5 RBCs/HPF or CD-H >12 months.

The primary outcome was relapse. We evaluated time to first event for relapse with start date defined as the time a patient first attained PR. As an exploratory study, the association between worsening hematuria and short-term (within 12 months) relapse was also analyzed. The worsening hematuria was defined as the appearance of positive hematuria (>3 RBCs/HPF). In this part, the exposures were the appearance of positive hematuria within 12 months before relapse (vs. no appearance) and the specific time frames (concurrent with relapse, over the past 3 months, over the past 4 to 6 months, or over the past 7 to 12 months) that the positive hematuria appeared in.

The secondary outcome was renal progression, which was a composite endpoint that included a ≥40% decline in eGFR or ESRD. The time zero was kidney biopsy. For futher research, the relationship of hematuria remission with renal progression was also quantified. Hematuria remission was defined by the absence of microhematuria or the presence of ≤3 RBCs/HPF in all urine sediment tests performed during at least 12 months before the last outpatient visit. More details on the methods are provided in the supplementary material (Supplementary Appendix 1, Supplementary Table 1).

### Statistical Analysis

Continuous variables are presented as means with standard deviations (SD) for normal distributions or medians with interquartile range (IQR) for skewed distributions. Categorical variables are presented as percentages. Differences between groups were compared using Student's *t*-tests for normally distributed variables, Kolmogorov-Smirnov tests for variables with skewed distributions, and chi-squared tests for categorical variables. The cumulative probability of relapse was estimated using the Kaplan-Meier method and compared using the log-rank test. Adjusted analyses were performed using Cox proportional hazards regression. The proportionality assumption of models was evaluated *via* the examination of Shoenfeld residuals. For renal progression, the outcome probability was evaluated using univariate analyses with cumulative incidence function (CIF) methods and Gray test and multivariate competing risk regression analyses. Death without renal progression was treated as a competing event. TA-H, CD-H, and TA-P were included as time-varying covariates. Initial hematuria, TA-H, CD-H, and TA-P were log-transformed due to their positively skewed distribution. To avoid data loss due to transformation, 0.1 was added to the values of initial hematuria, TA-H, and CD-H. The strength of association is expressed as a hazard ratio (HR) or subdistribution hazard ratio (sHR) with 95% confidence intervals (95% CIs).

Sensitivity analyses were performed by (1) restricting the subcohort to a single center or (2) using a 50% decline in renal function or ESRD as the endpoint. We also tested whether nephrotic syndrome (vs. subnephrotic status) and the use of RAAS blockers or IS agents modified the effect of initial hematuria or hematuria classification by adding the interaction terms in the models. A two-sided *P* value <0.05 was considered significant. Statistical analyses were performed using Stata Edition 15.0 (StataCorp., College Station, TX, USA).

## Results

### Patients and Outcomes

**Figure 1:**
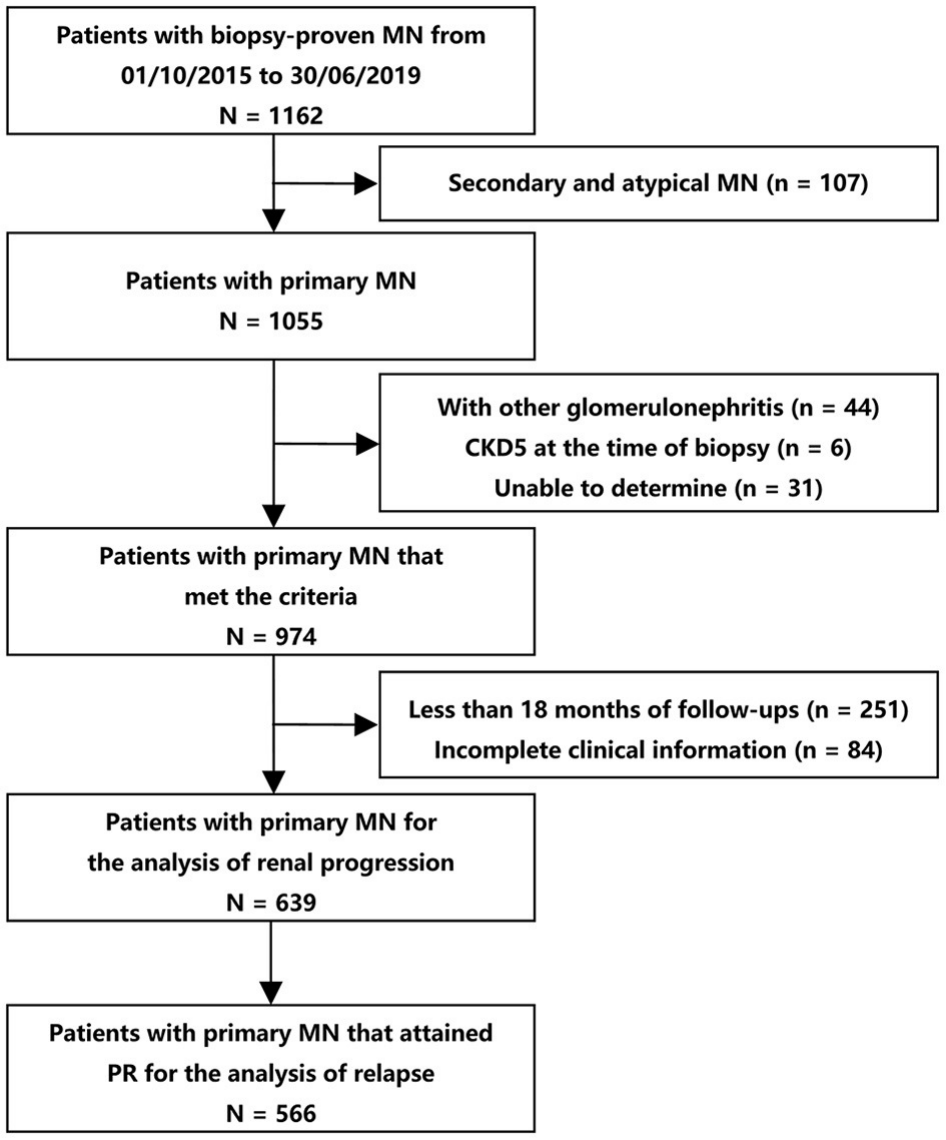
Flow diagram of patient selection. The patients that reached PR (*N* = 566) were used for the analyses of the association between initial hematuria and relapse. All the eligible patients (*N* = 639) were included for the analyses of the association between microhematuria and kidney disease progression. CKD, chronic kidney disease; MN, membranous nephropathy; PR, partial remission.

Among 1,162 participants from the two centers, 639 were eligible for analyses (474 from XH and 165 from SPHTCM) (Figure 1). At baseline, data were missing for 4.69% (30/639) and 0.78% (5/639) of patients for body mass index and microhematuria, respectively. Participant clinical characteristics are described in Table 1. There were 409 (64.01%) males, and the median age was 49 (IQR, 37–59) years. A total of 236 (36.93%) patients had hypertension, and 311 (48.67%) presented with nephrotic syndrome. The mean eGFR was 95.92 ± 20.54 mL/min/1.73 m^2^, and the serum albumin was 2.88 ± 0.76 g/dL. The median proteinuria was 3.33 (1.75–5.10) g/d, and the microhematuria was 3 (1–8) RBCs/HPF at baseline.

**Table 1:** Characteristics of patients with primary membranous nephropathy.

**Variable**	**Total**	**TA-H**		***P*** **value**	**CD-H**		***P*** **value**
		**>5 RBCs/HPF**	**≤5 RBCs/HPF**		**>12 months**	**≤12 months**	
No. of patients	639	93	546		95	544	
**Baseline**							
Age (yr)	49 (37–59)	49 (38–58)	49 (37–59)	0.961	53 (41–62)	49 (36–58)	0.053
Males, *n* (%)	409 (64.01)	70 (75.27)	339 (62.09)	0.014	68 (71.58)	341 (62.68)	0.096
BMI (kg/m^2^)	24.6 (22.2–26.9)	24.6 (22.7–26.6)	24.6 (22.2–27.0)	0.747	24.6 (22.3–27.3)	24.5 (22.2–26.8)	0.910
Hypertension, *n* (%)	236 (36.93)	31 (33.33)	205 (37.55)	0.437	36 (37.89)	200 (36.76)	0.833
Nephrotic syndrome, *n* (%)	311 (48.67)	53 (56.99)	258 (47.25)	0.082	48 (50.53)	263 (48.35)	0.695
Serum creatine, mg/dL	0.87 ± 0.23	0.87 ± 0.20	0.87 ± 0.23	0.728	0.88 ± 0.26	0.86 ± 0.22	0.403
eGFR (ml/min per 1.73 m^2^)	95.92 ± 20.54	96.69 ± 20.80	95.79 ± 20.51	0.695	94.35 ± 22.44	96.19 ± 20.20	0.419
Serum albumin (g/dL)	2.88 ± 0.76	2.59 ± 0.75	2.93 ± 0.75	0.001	2.73 ± 0.73	2.90 ± 0.76	0.035
Microhematuria (RBCs/HPF)	3 (1–8)	7 (3–19)	3 (1–7)	<0.001	6 (3–18)	3 (1–7)	<0.001
Proteinuria (g/d)	3.33 (1.75–5.1)	3.76 (2.13–6.4)	3.18 (1.7–4.9)	0.196	3.51 (1.83–5.11)	3.29 (1.72–5.06)	0.675
**Follow-up**							
Follow-up duration (months)	40 (28–49)	31 (24–47)	41 (29–50)	0.001	42 (27–50)	40 (28–49)	0.591
Initial hematuria (RBCs/HPF)	3.20 (2.00–6.23)	8.57 (3.67–13.75)	2.98 (1.67–5.00)	<0.001	7.00 (3.20–11.00)	3.00 (1.73–5.08)	<0.001
TA-H (RBCs/HPF)	2.34 (1.28–3.78)	7.43 (5.98–8.83)	2.1 (1.14–2.94)	<0.001	6.00 (4.27–8.55)	2.10 (1.14–2.99)	<0.001
CD-H (months)	3 (1–8)	15 (9–23)	2 (1–6)	<0.001	19 (16–25)	2 (1–5)	<0.001
TA-P (g/d)	1.19 (0.65–2.07)	1.85 (1.19–3.05)	1.09 (0.61–1.91)	<0.001	2.10 (1.32–3.44)	1.09 (0.61–1.83)	<0.001
RAAS blockades, *n* (%)	496 (77.62)	63 (67.74)	433 (79.30)	0.013	67 (70.53)	429 (78.86)	0.072
IS agents				0.059			0.015
Monotherapy, *n* (%)	93 (14.55)	20 (21.51)	73 (13.37)		23 (24.21)	70 (12.87)	
Combination therapy, *n* (%)	434 (67.92)	62 (66.67)	372 (68.13)		58 (61.05)	376 (69.12)	
**Outcome**							
No remission, *n* (%)	73 (11.42)	33 (35.48)	40 (7.33)	<0.001	35 (36.84)	38 (6.99)	<0.001
Partial remission, *n* (%)	566 (88.56)	60 (64.52)	506 (92.67)	<0.001	60 (63.16)	506 (93.01)	<0.001
Relapse, *n* (%)	111 (19.61)	33 (55.00)	78 (15.42)	<0.001	33 (55.00)	78 (15.42)	<0.001
Complete remission, *n* (%)	385 (68.02)	34 (56.67)	351 (69.78)	0.046	33 (55.00)	352 (69.57)	0.022
Renal progression^a^, *n* (%)	50 (7.82)	14 (15.05)	36 (6.59)	0.007	18 (18.95)	32 (5.88)	<0.001
ESRD, *n* (%)	9 (1.41)	4 (4.30)	5 (0.92)	0.010	7 (7.37)	2 (0.37)	<0.001

*BMI, body mass index; CD-H, cumulative duration of hematuria; eGFR, estimated glomerular filtration rate; ESRD, end-stage renal disease; HPF, high-power field; IS agents, immunosuppressive agents; RAAS, renin-angiotensin-aldosterone system; RBCs, red blood cell counts; TA-H, time-average hematuria; TA-P, time-average proteinuria*.

a *Renal progression was defined as a 40% decline in the eGFR or ESRD*.

The median follow-up was 40 (28–49) months. The median initial hematuria was 3.2 (2–6.23) RBCs/HPF. The median TA-H was 2.34 (1.28–3.78) RBCs/HPF, CD-H was 3 (1–8) months, and TA-P was 1.19 (0.65–2.07) g/d. During follow-up, 566 (88.58%) patients in the entire cohort achieved PR. Among patients who reached PR, 111 (19.61%) subsequently relapsed in a median of 30 (18–41) months. Overall, 50 (7.82%) patients reached renal progression, including 9 ESRD events.

### Microhematuria and Relapse

**Figure 2:**
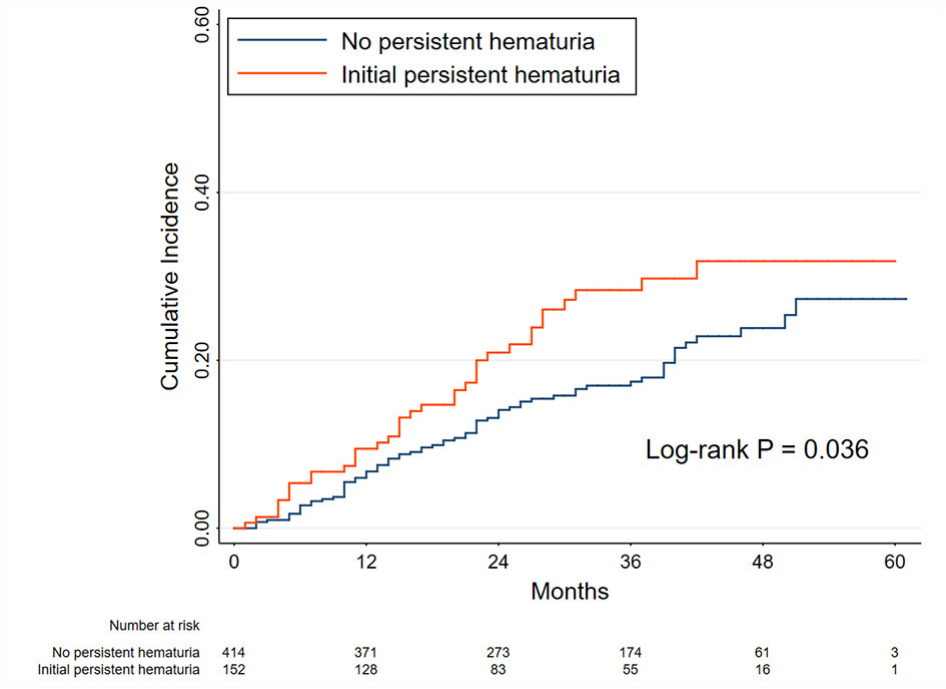
Kaplan-Meier curves for relapse in patients with and without initial persistent hematuria. The initial hematuria was defined as the average hematuria of the first 6 months of follow-up in patients with primary membranous nephropathy. The initial persistent hematuria was defined as an initial hematuria >5 RBCs/HPF. Between-group comparison was performed using the log-rank test. The time zero was when a person first attained partial remission.

The patients who attained PR (566 patients) were used for the analyses of this part (Figure 1 and Supplementary Table 1). The exposure was the initial hematuria. Figure 2 shows the Kaplan-Meier curves for relapse in PMN patients with and without initial persistent hematuria. The 3-year and 5-year cumulative probabilities of relapse were 28.36 and 31.81%, respectively, in patients with initial persistent hematuria, and 17.46 and 27.31%, respectively, in patients without initial persistent hematuria (*P* = 0.036).

The results of Cox proportional hazards regression analyses are presented in Table 2 and Supplementary Table 2. A higher level of initial hematuria was an independent risk factor for relapse (adjusted HR, 1.43; 95% CI, 1.15–1.78) in Model C after adjustment for age, sex, hypertension, serum albumin, eGFR, proteinuria, and use of RAAS blockers and IS agents. As a categorical variable, initial persistent hematuria was associated with a 1.52 (1.02–2.29) greater hazard of relapse in reference to patients without initial persistent hematuria.

**Table 2:** Microhematuria and risk of relapse in cox proportional hazards models.

**Factor**	**Hazard ratio for relapse (95% CI);** ***P*** **value**			
	**Unadjusted**	**Model A**	**Model B**	**Model C**
**Initial hematuria** (per 1 unit greater)	1.44 (1.17–1.79); 0.001	1.46 (1.18–1.81); <0.001	1.44 (1.16–1.79); 0.001	1.43 (1.15–1.78); 0.001
**Initial persistent hematuria** (in reference to no persistent hematuria)	1.52 (1.02–2.25); 0.038	1.58 (1.06–2.35); 0.024	1.54 (1.03–2.29); 0.036	1.52 (1.02–2.29); 0.042

*The initial hematuria, defined as the average hematuria of the first 6 months, was log-transformed. To avoid data loss, 0.1 was added to the value of initial hematuria. The initial persistent hematuria, defined as an initial hematuria value >5 RBCs/HPF, was expressed as a binary variable. Model A was adjusted for age, sex, and hypertension; sex and hypertension were expressed as categorical variables. Model B was adjusted for covariates in Model A plus baseline serum albumin, eGFR, and proteinuria. Model C was adjusted for covariates in Model B plus use of RAAS blockers and IS agents. Use of RAAS blockers and IS agents were expressed as categorical variables*.

*CI, confidence interval; eGFR, estimated glomerular filtration rate; IS agents, immunosuppressive agents; RAAS, renin-angiotensin-aldosterone system*.

The effect size was recalculated in a single center in sensitivity analyses. The adjusted HR of initial hematuria in the XH center (Supplementary Table 3) was 1.49 (1.15–1.94) in Model C. The corresponding adjusted HR was 1.57 (1.00–2.48) in the SPHTCM center (Supplementary Table 4). Between the nephrotic and subnephrotic groups, no heterogeneity was observed for the association of initial hematuria with relapse (*P* for interaction = 0.471). Neither the use of RAAS blockers (*P* for interaction = 0.397) nor IS agents (*P* for interaction = 0.850) significantly modified the effect of initial hematuria.

### Worsening Hematuria and Short-Term Relapse

In this part, the patients that reached PR (566 patients) were used for the analyses (Figure 1 and Supplementary Table 1). As shown in Table 3 and Supplementary Table 5, the appearance of positive hematuria substantially enhanced the risk of short-term relapse, in reference to no appearance, after adjustment for negative conversion of hematuria and other confounders (adjusted HR, 4.64; 3.29–6.54). Compared to a flare concurrent with hematuria, worsening hematuria was associated with a higher risk of relapse within the next 4–6 months (adjusted HR, 1.57; 1.04–2.38).

**Table 3:** Worsening hematuria and risk of short-term relapse in cox proportional hazards models.

**Factor**	**Hazard ratio for relapse (95% CI);** ***P*** **value**			
	**Unadjusted**	**Model A**	**Model B**	**Model C**
**Appearance of positive hematuria** (vs. no appearance)	3.83 (2.76–5.33); <0.001	4.66 (3.31–6.56); <0.001	4.66 (3.31–6.55); <0.001	4.64 (3.29–6.54); <0.001
**Time of hematuria appearance**				
Concurrent with relapse	1.00 (Reference)	1.00 (Reference)	1.00 (Reference)	1.00 (Reference)
Over past 3 mo	0.92 (0.65–1.29); 0.627	1.14 (0.80–1.62); 0.466	1.10 (0.77–1.57); 0.589	1.12 (0.78–1.59); 0.536
Over past 4 to 6 mo	0.91 (0.66–1.25); 0.560	1.48 (0.98–2.21); 0.060	1.51 (1.00–2.26); 0.048	1.57 (1.04–2.38); 0.031
Over past 7 to 12 mo	0.76 (0.58–0.98); 0.034	1.15 (0.81–1.63); 0.426	1.12 (0.79–1.59); 0.534	1.13 (0.80–1.61); 0.492

*Worsening hematuria and negative conversion of hematuria were included as time-varying covariates. Model A was adjusted for negative conversion of hematuria, age, sex, and hypertension; negative conversion of hematuria, sex and hypertension were expressed as categorical variables. Model B was adjusted for covariates in Model A plus baseline serum albumin, baseline eGFR, and proteinuria. Model C was adjusted for covariates in Model B plus RAAS blockers and IS agents. Use of RAAS blockers and IS agents were expressed as categorical variables*.

*CI, confidence interval; eGFR, estimated glomerular filtration rate; IS agents, immunosuppressive agents; RAAS, renin-angiotensin-aldosterone system; TA-P, time-averaged proteinuria*.

### Microhematuria and Renal Progression

All eligible participants (639 patients) were included in the analysis of the association of microhematuria with renal progression (Figure 1 and Supplementary Table 1). The exposures were the initial hematuria, TA-H, and CD-H. According to the magnitude of microhematuria over time, 15.05% (14/93) and 6.59% (36/546) of patients with PMN reached composite renal progression events, respectively, in the persistent hematuria (TA-H >5 RBCs/HPF) and non-persistent hematuria (TA-H ≤5 RBCs/HPF) groups. As for the persistence of microhematuria, 18.95% (18/95) and 5.88% (32/544) of patients reached composite renal progression events, respectively, in the persistent hematuria (CD-H >12 months) and non-persistent hematuria (CD-H ≤12 months) groups (Table 1).

**Figure 3:**
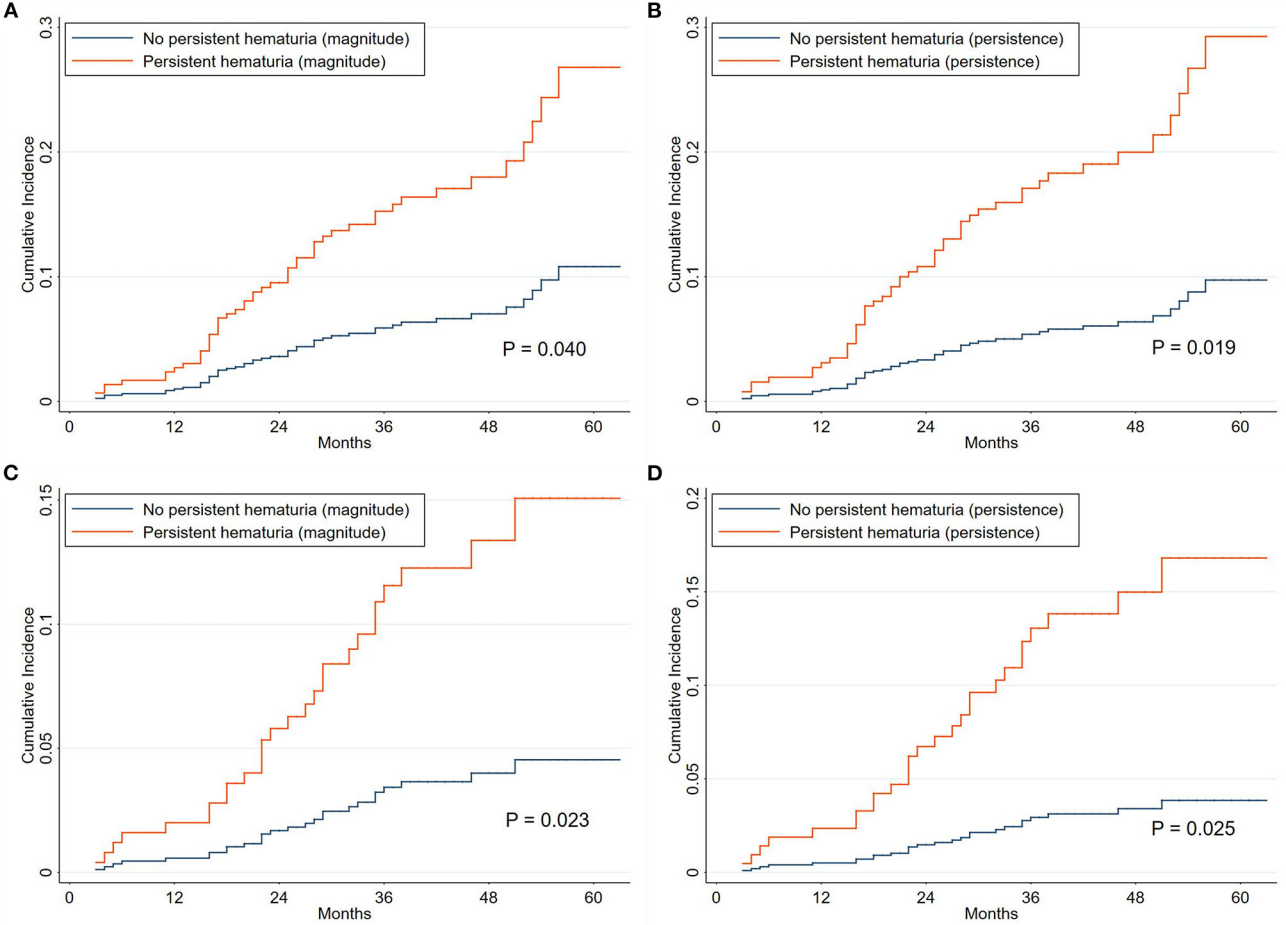
Cumulative incidence curves for composite renal progression events grouped by hematuria classification. The cumulative incidence function methods and Gray test were used for analyses. The time zero was kidney biopsy. According to the magnitude of microhematuria over time, the persistent hematuria was defined as time-averaged hematuria >5 RBCs/HPF **(A,C)**. As for the persistence of microhematuria, the persistent hematuria was defined as cumulative duration of hematuria >12 months **(B,D)**. The corresponding endpoints were 40% decline in renal function or end-stage renal disease **(A,B)** and 50% decline in renal function or end-stage renal disease **(C,D)**. Death without renal progression was treated as a competing event.

As shown in Table 4 and Supplementary Table 6, initial hematuria might not be associated with renal progression (adjusted sHR, 1.25; 0.93–1.67), but TA-H (adjusted sHR, 1.35; 1.12–1.63) and CD-H (adjusted sHR, 1.17; 1.02–1.34) were independent predictors of renal progression after adjustment for age, sex, hypertension, serum albumin, eGFR, proteinuria, and use of RAAS blockers and IS agents in the competing risk regression Model C. According to the magnitude of microhematuria over time, persistent hematuria was associated with a greater risk of renal progression (*P* = 0.040, Figure 3A). As for the persistence of microhematuria, similar result was observed (*P* = 0.019, Figure 3B).

**Table 4:** Microhematuria and risk of renal progression in competing risk regression models.

**Factor**	**Subdistribution hazard ratio for renal progression (95% CI);** ***P*** **value**			
	**Unadjusted**	**Model A**	**Model B**	**Model C**
**Initial hematuria** (per 1 unit greater)	1.37 (1.08–1.74); 0.009	1.38 (1.08–1.76); 0.009	1.26 (0.95–1.67); 0.104	1.25 (0.93–1.67); 0.137
**Initial persistent hematuria** (in reference to no persistent hematuria)	2.33 (1.34–4.05); 0.003	2.43 (1.38–4.27); 0.002	2.19 (1.19–4.05); 0.012	2.19 (1.17–4.12); 0.015
**TA-H** (per 1 unit greater)	1.45 (1.23–1.69); <0.001	1.47 (1.25–1.74); <0.001	1.34 (1.11–1.61); 0.002	1.35 (1.12–1.63); 0.002
**Persistent hematuria based on TA-H** (in reference to no persistent hematuria)	2.73 (1.47–5.05); 0.001	2.87 (1.55–5.32); 0.001	2.07 (1.07–4.01); 0.030	2.01 (1.03–3.92); 0.040
**CD-H** (per 1 unit greater)	1.25 (1.09–1.44); 0.001	1.24 (1.09–1.41); 0.001	1.17 (1.03–1.34); 0.020	1.17 (1.02–1.34); 0.021
**Persistent hematuria based on CD-H** (in reference to no persistent hematuria)	3.38 (1.91–5.99); <0.001	3.12 (1.75–5.55); <0.001	2.24 (1.15–4.35); 0.018	2.22 (1.14–4.33); 0.019

*Renal progression was defined as a 40% decline in the eGFR or ESRD. Death without renal progression was treated as a competing event. TA-H, CD-H, and TA-P were included as time-varying covariates. Initial hematuria, TA-H, CD-H, and TA-P were log-transformed due to their positively skewed distribution. To avoid data loss due to transformation, 0.1 was added to the values of initial hematuria, TA-H, and CD-H. Initial persistent hematuria was defined by initial hematuria >5 RBCs/HPF. Persistent hematuria was defined by TA-H >3 RBCs/HPF or CD-H >12 months. The initial persistent hematuria and persistent hematuria were expressed as binary variables. Model A was adjusted for age, sex, and hypertension; sex and hypertension were expressed as categorical variables. Model B was adjusted for covariates in Model A plus baseline serum albumin, baseline eGFR, and TA-P. Model C was adjusted for covariates in Model B plus use of RAAS blockers and IS agents. Use of RAAS blockers and IS agents were expressed as categorical variables*.

*CD-H, cumulative duration of hematuria; CI, confidence interval; eGFR, estimated glomerular filtration rate; ESRD, end-stage renal disease; IS agents, immunosuppressive agents; RAAS, renin-angiotensin-aldosterone system; TA-H, time-averaged hematuria; TA-P, time-averaged proteinuria*.

The clinical characteristics of the patients grouped by centers are presented in Supplementary Table 7. The results of sensitivity analyses from each center were consistent with the entire cohort. The adjusted sHRs in the XH center (Supplementary Table 8) were 1.55 (1.21–1.98) and 1.27 (1.09–1.48) for TA-H and CD-H, respectively. The corresponding adjusted sHRs in the SPHTCM center (Supplementary Table 9) were 1.12 (0.82–1.54) and 1.04 (0.90–1.20), respectively. The results were consistent when the endpoint was a 50% decline in renal function or ESRD, and the corresponding adjusted sHRs were 1.38 (1.12–1.71) and 1.23 (1.00–1.51), respectively, for TA-H and CD-H (Figures 3C,D and Supplementary Table 10). Interaction analyses also showed no significant heterogeneity of the association between TA-H and renal progression (nephrotic syndrome, *P* for interaction = 0.868; use of RAAS blockers, *P* for interaction = 0.824; use of IS agents, *P* for interaction = 0.405). Similarly, no significant heterogeneity was observed in the analyses of the association between CD-H and renal progression (nephrotic syndrome, *P* for interaction = 0.869; use of RAAS blockers, *P* for interaction = 0.495; use of IS agents, *P* for interaction = 0.288).

### Hematuria Remission and Renal Progression

**Figure 4:**
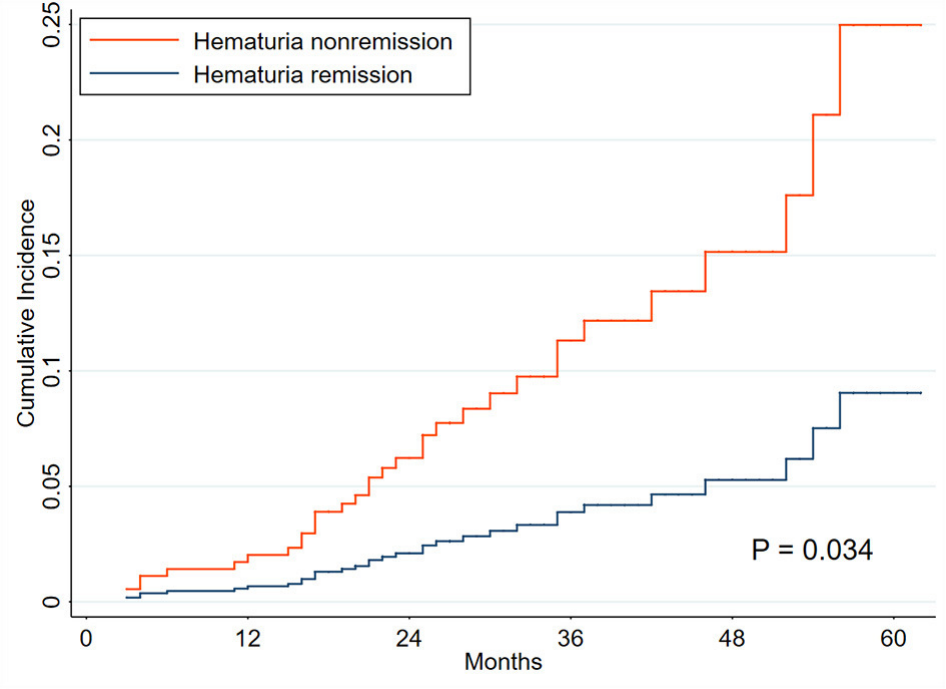
Cumulative incidence curves for composite renal progression events grouped by hematuria remission. The cumulative incidence function methods and Gray test were used for analyses. The time zero was kidney biopsy. The endpoint was renal progression, defined as a 40% decline in renal function or end-stage renal disease. Death without renal progression was treated as a competing event.

Patients with positive hematuria (TA-H >3 RBCs/HPF, 220 patients) were used for the analyses, and were divided into hematuria remission (71 patients) and non-remission (149 patients) groups (Supplementary Table 1). The exposure was the hematuria remission, which was clearly defined in the methods. Five (7.04%) patients in the remission group and 28 (18.79%) patients in the non-remission group reached composite renal progression events (Supplementary Table 11). Hematuria remission, included as a time-varying covariate, was substantially associated with a reduced risk of renal progression in Model C (adjusted sHR, 0.63; 0.41–0.96) (Table 5 and Supplementary Table 12). CIF analysis revealed that the incidence of renal progression considerably decreased in the remission group compared to the non-remission group (*P* = 0.034, Figure 4).

**Table 5:** Hematuria remission and risk of renal progression.

**Factor**	**Sub-distribution hazard ratio for renal progression (95% CI);** ***P*** **value**			
	**Unadjusted**	**Model A**	**Model B**	**Model C**
**Hematuria remission**	0.61 (0.40–0.93); 0.023	0.63 (0.41–0.96); 0.033	0.64 (0.41–0.98); 0.040	0.63 (0.41–0.96); 0.034

*Renal progression was defined as a 40% decline in eGFR or ESRD. Death without renal progression was treated as a competing event. Hematuria remission, included as a time-varing covariate, was the absence of hematuria or the presence of ≤3 RBCs/HPF in all the urine sediment tests performed during at least 12 months before the last outpatient visit. Model A was adjusted for age, sex, and hypertension; sex and hypertension were expressed as categorical variables. Model B was adjusted for covariates in Model A plus baseline serum albumin, baseline eGFR, and TA-P; TA-P was log-transformed and included as a time-varying covariate. Model C was adjusted for covariates in Model B plus RAAS blockades and IS agents. Use of RAAS blockades and IS agents were expressed as categorical variables*.

*CI, confidence interval; eGFR, estimated glomerular filtration rate; ESRD, end-stage renal disease; HPF, high power field; IS agents, immunosuppressive agents; RAAS, renin-angiotensin-aldosterone system; TA-P, time-averaged proteinuria*.

## Discussion

Taken together, the results from our double-center cohort suggested that (1) the initial hematuria was an independent risk factor for relapse, and the magnitude and persistence of microhematuria over time were substantially associated with kidney disease progression in patients with PMN; (2) the worsening of microhematuria markedly enhanced the hazard of short-term relapse; and (3) the remission of microhematuria exhibited a positive effect on the improvement in renal outcomes in patients with PMN and microhematuria during follow-up.

Hematuria is a clinical symptom of some renal disorders with benign features, and it has been neglected by many clinicians for decades. However, multiple recent studies challenged this concept and demonstrated that the presence of hematuria, especially of glomerular origin, was a hallmark of inflammation and pathological damage in nephridial tissue (4–6, 23). The accumulation of heavy microhematuria over time may be a predictor of adverse outcomes, e.g., disease activity in systemic lupus erythematosus (24, 25), renal relapse in AAV (10, 26, 27), and kidney disease progression in IgAN (7–9).

The most prominent clinical feature of PMN is nephrotic syndrome and its associated manifestations. Microhematuria is not uncommon and accounts for ~50% of patients at disease onset and 60% during the course (11–13, 15, 18, 28, 29). However, few studies focused on the prognostic relevance of the magnitude and persistence of microhematuria over time in PMNs. A total of 316 (49.45%) patients in our double-center cohort had urinary erythrocyte counts of >3 RBCs/HPF at baseline, and 220 (34.43%) patients had positive hematuria (TA-H >3 RBCs/HPF) during follow-up.

Previous studies generally used TA-H >5 RBCs/HPF to represent persistent hematuria (8, 9). However, 37 patients in our cohort had TA-H values >5 RBCs/HPF but a CD-H <12 months, and 8 patients had a CD-H <6 months. Obviously, the application of the previous definition in these patients would not objectively reflect the real status of hematuria during the disease course. Our study quantified the magnitude and persistence of microhematuria over time using TA-H and CD-H, respectively.

Our results suggested that the initial persistent hematuria was independently associated with a greater risk of a future flare in patients with PMN. One of the greatest future challenges is the establishment of a clear association between glomerular damage and the potential utility of initial persistent hematuria to decide when it is necessary to increase IS treatment. Moreover, there was an obvious connection between worsening hematuria and the risk of a flare within the following 12 months. The appearance of positive hematuria was related to an increased risk of relapse in reference to no appearance. Notably, the worsening of hematuria over the past 4–6 months presented a more prominent warning compared to the occurrence of hematuria at the onset of a relapse. These findings support the routine use of urine sediments in clinical practice and emphasize the significance of close clinical monitoring in patients with PMN because this may be an early predictor of short-term relapse.

The hazards of kidney disease progression in PMN patients with persistent hematuria was significantly higher than those with non-persistent hematuria during followup. It has been widely accepted that glomerular hematuria resulted from the passage of erythrocytes from the glomerular capillary into the urinary space and was associated with glomerular filtration barrier damage. In primary glomerulonephritis or autoimmune diseases, infiltrating leukocytes may release metalloproteinases and reactive oxygen species, leading to a glomerular basement membrane which is more susceptible to rupture (5, 30). Futhermore, persistent glomerular hematuria might represent a continued “low-grade” activity of the underlying inflammatory process. This could also induce renal damage *via* the oxidative stress caused by the release of hemoglobin and iron from broken erythrocytes into renal tubular cells (5, 8, 30–34). On the other hand, the remission of hematuria independently reduced the hazard of renal progression by 37% in patients with positive microhematuria. The search for adequate surrogate markers of kidney disease progression is a key issue in many clinical conditions (5, 16). Therefore, we suggest that hematuria remission may be a surrogate marker of renal survival for patients with PMN and microhematuria. However, the relationship between hematuria remission and hard endpoints must be confirmed in prospective and long-term cohorts.

Our study has the following strengths. (1) This was the first study to investigate the prognostic relevance of microhematuria over time in PMN. (2) The magnitude and persistence of microhematuria over time were systematically quatified. (3) The relationships between the worsening hematuria and short-term relapse, and between hematuria remission and kidney disease progression were also analyzed. (4) The two-center design made it possible to validate the robustness of our findings.

Our study is not without its limitations. (1) This study was a retrospective cohort with a single ethnicity. (2) We did not include comprehensive data that may be associated with renal survival in patients with PMN, such as serum anti-PLA_2_R levels after therapy, urinary excretion of β^2^ microglobulin, and other known or unknown confounders. Therefore, we cannot exclude the possibility of residual confounding. (3) The relatively low number of renal progression events may affect the accuracy of the results.

In summary, our study showed that initial hematuria was an independent predictor for relapse, and the magnitude and persistence of microhematuria over time were independently linked with a higher risk of renal function loss in patients with PMN. Further studies are needed to verify our findings and clarify the role of surveillance and treatment of hematuria in clinical management decisions for PMN.

## Data Availability Statement

The raw data supporting the conclusions of this article will be made available by the authors, without undue reservation.

## Ethics Statement

The studies involving human participants were reviewed and approved by the Ethics Committee of Xijing Hospital. Written informed consent from the participants' legal guardian/next of kin was not required to participate in this study in accordance with the national legislation and the institutional requirements.

## Author Contributions

LH, PH, SS, CH, and HW: designed the study, analyzed the data, and drafted the manuscript. PH, JL, and YZ: collected and entered data. LH, PH, XY, SS, CH, and HW: contributed to data acquisition and interpretation. All authors have read and approved the final manuscript.

## Funding

This study was supported in part by grants from the National Natural Science Foundation of China (81770764 and 81770669).

## Conflict of Interest

The authors declare that the research was conducted in the absence of any commercial or financial relationships that could be construed as a potential conflict of interest.

## Publisher's Note

All claims expressed in this article are solely those of the authors and do not necessarily represent those of their affiliated organizations, or those of the publisher, the editors and the reviewers. Any product that may be evaluated in this article, or claim that may be made by its manufacturer, is not guaranteed or endorsed by the publisher.

## References

[1] Kidney Disease Improving Global Outcomes (KDIGO) Glomerulonephritis Work Group: KDIGO clinical practice guideline for glomerulonephritis. Kidney Int Suppl. (2012) 2:139–274.

[2] Cybulsky AV Walsh M Knoll G Hladunewich M Bargman J Reich H. Canadian society of nephrology commentary on the 2012. KDIGO clinical practice guideline for glomerulonephritis: management of glomerulonephritis in adults. Am J Kidney Dis. (2014) 63:363–77. 10.1053/j.ajkd.2013.12.001 24423780

[3] Beck L Bomback AS Choi MJ Holzman LB Langford C Mariani LH. KDOQI US commentary on the 2012. KDIGO clinical practice guideline for glomerulonephritis. Am J Kidney Dis. (2013) 62:403–41. 10.1053/j.ajkd.2013.06.002 23871408

[4] Peterson LM Reed HS. Hematuria. Prim Care. (2019) 46:265–73. 10.1016/j.pop.2019.02.008 31030828

[5] Moreno JA Sevillano A Gutierrez E Guerrero-Hue M Vazquez-Carballo C Yuste C. Glomerular hematuria: cause or consequence of renal inflammation? Int J Mol Sci. (2019) 20:19. 10.3390/ijms20092205 31060307 PMC6539976

[6] Coppo R Fervenza FC. Persistent microscopic hematuria as a risk factor for progression of iga nephropathy: new floodlight on a nearly forgotten biomarker. J Am Soc Nephrol. (2017) 28:2831–4. 10.1681/ASN.2017060639 28739649 PMC5619979

[7] Le W Liang S Hu Y Deng K Bao H Zeng C. Long-term renal survival and related risk factors in patients with IgA nephropathy: results from a cohort of 1155 cases in a Chinese adult population. Nephrol Dial Transplant. (2012) 27:1479–85. 10.1093/ndt/gfr527 21965586

[8] Sevillano AM Gutierrez E Yuste C Cavero T Merida E Rodriguez P. Remission of hematuria improves renal survival in Iga nephropathy. J Am Soc Nephrol. (2017) 28:3089–99. 10.1681/ASN.2017010108 28592423 PMC5619972

[9] Yu GZ Guo L Dong JF Shi SF Liu LJ Wang JW. Persistent hematuria and kidney disease progression in Iga nephropathy: a cohort study. Am J Kidney Dis. (2020) 76:90–99. 10.1053/j.ajkd.2019.11.008 32197881

[10] Rhee RL Davis JC Ding L Fervenza FC Hoffman GS Kallenberg C. The utility of urinalysis in determining the risk of renal relapse in anca-associated vasculitis. Clin J Am Soc Nephrol. (2018) 13:251–7. 10.2215/CJN.04160417 29371340 PMC5967421

[11] Alsharhan L Beck LJ. Membranous nephropathy: core curriculum. Am J Kidney Dis. (2021) 77:440–53. 10.1053/j.ajkd.2020.10.009 33487481

[12] Couser WG. Primary membranous nephropathy. Clin J Am Soc Nephrol. (2017) 12:983–97. 10.2215/CJN.11761116 28550082 PMC5460716

[13] Cattran DC Brenchley PE. Membranous nephropathy: integrating basic science into improved clinical management. Kidney Int. (2017) 91:566–74. 10.1016/j.kint.2016.09.048 28065518

[14] Ronco P Debiec H. Pathophysiological advances in membranous nephropathy: time for a shift in patient's care. Lancet. (2015) 385:1983–92. 10.1016/S0140-6736(15)60731-0 26090644

[15] Ponticelli C Glassock RJ. Glomerular diseases: membranous nephropathy–a modern view. Clin J Am Soc Nephrol. (2014) 9:609–16. 10.2215/CJN.04160413 23813556 PMC3944756

[16] Thompson A Cattran DC Blank M Nachman PH. Complete and partial remission as surrogate end points in membranous nephropathy. J Am Soc Nephrol. (2015) 26:2930–7. 10.1681/ASN.2015010091 26078365 PMC4657845

[17] Hladunewich MA Troyanov S Calafati J Cattran DC. The natural history of the non-nephrotic membranous nephropathy patient. Clin J Am Soc Nephrol. (2009) 4:1417–22. 10.2215/CJN.01330209 19661220 PMC2736692

[18] Barbour S Reich H Cattran D. Short-term complications of membranous nephropathy. Contrib Nephrol. (2013) 181:143–51. 10.1159/000349976 23689576

[19] De Vriese AS Glassock RJ Nath KA Sethi S Fervenza FC. A proposal for a serology-based approach to membranous nephropathy. J Am Soc Nephrol. (2017) 28:421–30. 10.1681/ASN.2016070776 27777266 PMC5280030

[20] Polanco N Gutierrez E Covarsi A Ariza F Carreno A Vigil A. Spontaneous remission of nephrotic syndrome in idiopathic membranous nephropathy. J Am Soc Nephrol. (2010) 21:697–704. 10.1681/ASN.2009080861 20110379 PMC2844306

[21] Cattran DC Kim ED Reich H Hladunewich M Kim SJ. Membranous nephropathy: quantifying remission duration on outcome. J Am Soc Nephrol. (2017) 28:995–1003. 10.1681/ASN.2015111262 27756808 PMC5328151

[22] Levey AS Stevens LA Schmid CH Zhang YL Castro AR Feldman HI. A new equation to estimate glomerular filtration rate. Ann Intern Med. (2009) 150:604–12. 10.7326/0003-4819-150-9-200905050-00006 19414839 PMC2763564

[23] Vivante A Afek A Frenkel-Nir Y Tzur D Farfel A Golan E. Persistent asymptomatic isolated microscopic hematuria in Israeli adolescents and young adults and risk for end-stage renal disease. JAMA. (2011) 306:729–36. 10.1001/jama.2011.1141 21846854

[24] Ding JY Ibanez D Gladman DD Urowitz MB. Isolated hematuria and sterile pyuria may indicate systemic lupus erythematosus activity. J Rheumatol. (2015) 42:437–40. 10.3899/jrheum.140415 25593226

[25] Ayoub I Birmingham D Rovin B Hebert L. Commentary on the current guidelines for the diagnosis of lupus nephritis flare. Curr Rheumatol Rep. (2019) 21:12. 10.1007/s11926-019-0809-x 30810824

[26] Lv L Chang DY Li ZY Chen M Hu Z Zhao MH. Persistent hematuria in patients with antineutrophil cytoplasmic antibody-associated vasculitis during clinical remission: chronic glomerular lesion or low-grade active renal vasculitis? BMC Nephrol. (2017) 18:354. 10.1186/s12882-017-0763-7 29207950 PMC5717828

[27] Mahoney SL Nachman PH. Persistent hematuria in ANCA vasculitis: ominous or innocuous? Clin J Am Soc Nephrol. (2018) 13:201–202. 10.2215/CJN.14101217 29371338 PMC5967444

[28] Zhang XD Cui Z Zhang MF Wang J Zhang YM Qu Z. Clinical implications of pathological features of primary membranous nephropathy. BMC Nephrol. (2018) 19:215. 10.1186/s12882-018-1011-5 30153817 PMC6114049

[29] Jiang Z Cai M Dong B Yan Y Yang B Wang M. Clinicopathological features of atypical membranous nephropathy with unknown etiology in adult Chinese patients. Medicine (Baltimore). (2018) 97:e11608. 10.1097/MD.0000000000011608 30095619 PMC6133607

[30] Moreno JA Yuste C Gutierrez E Sevillano AM Rubio-Navarro A Amaro-Villalobos JM. Haematuria as a risk factor for chronic kidney disease progression in glomerular diseases: a review. Pediatr Nephrol. (2016) 31:523–33. 10.1007/s00467-015-3119-1 25980470

[31] Deuel JW Schaer CA Boretti FS Opitz L Garcia-Rubio I Baek JH. Hemoglobinuria-related acute kidney injury is driven by intrarenal oxidative reactions triggering a heme toxicity response. Cell Death Dis. (2016) 7:e2064. 10.1038/cddis.2015.392 26794659 PMC4816175

[32] Yuste C Rubio-Navarro A Barraca D Aragoncillo I Vega A Abad S. Haematuria increases progression of advanced proteinuric kidney disease. PLoS ONE. (2015) 10:e0128575. 10.1371/journal.pone.0128575 26016848 PMC4446357

[33] Ryan M Ware K Qamri Z Satoskar A Wu H Nadasdy G. Warfarin-related nephropathy is the tip of the iceberg: direct thrombin inhibitor dabigatran induces glomerular hemorrhage with acute kidney injury in rats. Nephrol Dial Transplant. (2014) 29:2228–34. 10.1093/ndt/gft380 24009280

[34] Moreno JA Martin-Cleary C Gutierrez E Rubio-Navarro A Ortiz A Praga M. Haematuria: the forgotten CKD factor? Nephrol Dial Transplant. (2012) 27:28–34. 10.1093/ndt/gfr749 22287699

