# Supplementary material for: Microhematuria Enhances the Risks of Relapse and Renal Progression in Primary Membranous Nephropathy
Source: Front Med (Lausanne). 2021 Dec 9;8:704830. doi: 10.3389/fmed.2021.704830 (PMC8695761; doi:10.3389/fmed.2021.704830)
Supplement: Supplementary file 1 [file Data_Sheet_1.PDF]

Microhematuria Enhances the Risks of Relapse and Renal Progression in Primary Membranous Nephropathy

Peng He[1,†], Xiaoyong Yu[2,†], Yang Zha[1,†], Jing Liu[1,†], Hanmin Wang[1], Chen Huang[1], Shiren Sun[1], Lijie He[1,*].

[1]Department of Nephrology, Xijing Hospital, the Fourth Military Medical University, Xi'an, Shaan xi, 710032, China.

[2]Department of Nephrology, Shaanxi Traditional Chinese Medicine Hospital, No. 2 Xihuamen, Xi’an, Shaanxi, 710003, China.

*Correspondence should be addressed to helijie@fmmu.edu.cn (LJH)

[†]These authors contributed equally to this work.

Supplemental Material

Appendix S1. Additional Information on Methods

Table S1. Summary of the Information about the Study Design

Table S2. Microhematuria and Risk of Relapse in the Cox Proportional Hazards Model C

Table S3. Microhematuria and Risk of Relapse in the XH Center

Table S4. Microhematuria and Risk of Relapse in the SPHTCM Center

Table S5. Worsening Hematuria and Risk of Short-Term Relapse in Cox Proportional Hazards Model C

Table S6. Microhematuria and Risk of Renal Progression in Competing Risk Regression Model C

Table S7. Characteristics of Patients Grouped by Centers

Table S8. Microhematuria and Risk of Renal Progression in the XH Center

Table S9. Microhematuria and Risk of Renal Progression in the SPHTCM Center

Table S10. Microhematuria and Risk of 50% Decline in Renal Function or ESRD

Table S11. Characteristics of Patients Grouped by Hematuria Remission

Table S12. Hematuria Remission and Risk of Renal Progression in Competing Risk Regression Model C

Appendix S1. Additional Information on Methods

Patient Selection

The "sufficient information on treatments and laboratory parameters" mainly referred to the baseline (age, gender, blood pressure, serum creatine, serum albumin, urine sediment, 24-h proteinuria excretion, and medication) and follow-up data (serum creatine, serum albumin, urine sediment, and 24-h proteinuria excretion) of patients were available for statistical descriptions and analyses, and patients who had not received immunosuppressive therapy for an extended period (>3 month) prior to kidney biopsy.

Patients with secondary membranous nephropathy, atypical membranous nephropathy, or other concomitant glomerular diseases were not included our analyses. To be specific, patients with membranous nephropathy that associated with other diseases or exposures, such as infections, autoimmune diseases, malignancy, drugs/toxins, were diagnosed as secondary membranous nephropathy and excluded; atypical membranous nephropathy was identified by kidney biopsy; other glomerular disease involved IgA nephropathy, ANCA-associated vasculitis, lupus nephritis, and et al..

Data collection

We only recorded the results of urine sendiment analyses, mainly of glomerular derived RBCs (dysmorphic RBCs >70%). If the microscopic analyses showed that the RBCs were mainly monomorphic, the persons received ultrasonic examination or even endoscopy; thus, urologic causes of hematuria were excluded. Additionally, since our study was focused on the microhematuria, the results of microscopic analyses during gross hematuria episodes were excluded.

At baseline, data were missing for 4.69% of patients for body mass index, and data were missing for <1% for all other variables. During follow-up, visits with missing microscopic urinalysis and proteinuria data were imputed using last observation carried forward method. Patients who had missing data at more than 50% of visits were not included in the statistical analyses. For all analyses, complete case methods were used, for which each model analysis was limited to patients with complete data for all model variables.

Definitions

The cumulative duration of hematuria was defined as the total number of months with microscopic analyses of >3 RBCs/HPF. To be specific, the positive result (>3 RBCs/HPF) of a single microscopic test was counted as 1 month; the duration between two adjacent positive results was equal to the difference between the two dates plus 1 month. For example, the results and dates of one patient's tests were “4, 2021/1/25; 5, 2021/4/23”. According to our definitions, the cumulative duration in this period was 4 months (4-1+1=4). However, if the results were “4, 2021/1/25; 1, 2021/4/23; 5, 2021/7/23”, the cumulative duration was 2 months (1+1=2).

We evaluated time to first event for relapse with start date defined as the time a patient first attained partial remission (PR). PR was defined by a proteinuria of >0.3 but <3.5 g/d plus a ≥50% reduction from its baseline level, along with serum albumin≥3.5 g/dL and stable kidney function. For patients with subnephrotic proteinuria, the date of PR was identified by proteinuria<3.5 g/d with serum albumin≥3.5 g/dL and stable kidney function. According to our observations, in this cohort, all relapse events occurred before death, so we did not choose a competing risk regression model.

Table S1. Summary of the Information about the Study Design

Exposure	Endpoint	Study population (patient no.)	Start date	End date	Statistical method
Initial hematuria and initial persistent hematuria	Relapse	Patients that attained partial remission (566)	Partial remission	Relapse/last outpatient visit/ESRD/death	Cox regression
Worsening hematuria	Relapse	Patients that attained partial remission (566)	Partial remission	Relapse/last outpatient visit/ESRD/death	Cox regression
TA-H, CD-H, and hematuria classification	Renal progression	All the patients (639)	Renal biopsy	Renal progression/last outpatient visit/ESRD/death	Competing risk regression
Hematuria remission	Renal progression	Patients with TA-H>3 (220)	Renal biopsy	Renal progression/last outpatient visit/ESRD/death	Competing risk regression

ESRD, end stage renal disease; TA-H, time-averaged hematuria; CD-H, cumulative duration of hematuria

Table S2. Microhematuria and Risk of Relapse in the Cox Proportional Hazards Model C

Variable	Hazard Ratio	95% Confidence Interval	P Value
Initial hematuria	1.43	1.15-1.78	0.001
Age	1.01	0.99-1.02	0.489
Male gender	1.27	0.83-1.94	0.274
Hypertension	0.91	0.59-1.40	0.668
Albumin	0.99	0.73-1.34	0.926
Proteinuria	1.04	0.98-1.10	0.187
EGFR	1.00	0.99-1.02	0.475
IS agents treatment			
Monotherapy	2.16	1.02-4.60	0.045
Combination therapy	1.58	0.80-3.12	0.184
RAAS blockade treatment	1.04	0.64-1.69	0.873

Initial hematuria was log-transformed due to their positively skewed distribution. To avoid data loss due to transformation, 0.1 was added to the values of initial hematuria.

EGFR, estimated glomerular filtration rate; ESRD, end-stage renal disease; IS agents, immunosuppressive agents; RAAS, renin-angiotensin-aldosterone system.

Table S3. Microhematuria and Risk of Relapse in the XH Center

Factor	Hazard Ratio for Relapse (95% CI); *P* value			
	**Unadjusted**	**Model A**	**Model B**	**Model C**
**Initial hematuria** (per 1 unit greater)	1.52 (1.18-1.96); 0.001	1.54 (1.20-1.98); 0.001	1.51 (1.16-1.95); 0.002	1.49 (1.15-1.94); 0.003
**Initial persistent hematuria**	1.62 (1.05-2.50); 0.030	1.67 (1.08-2.59); 0.022	1.60 (1.03-2.49); 0.037	1.57 (1.00-2.45); 0.051

The initial hematuria, defined as the average hematuria of the first 6 months, was log-tranformed. In order to avoid data loss, 0.1 was added to the value of initial hematuria. The initial persistent hematuria, defined as a initial hematuria value of >5 RBCs/HPF, was expressed as a categorical variable. Model A was adjusted for age, sex, and hypertension; sex and hypertension were expressed as categorical variables. Model B was adjusted for covariates in Model A plus baseline serum albumin, eGFR, and proteinuria. Model C was adjusted for covariates in Model B plus use of RAAS blockades and IS agents. Use of RAAS blockades and IS agents were expressed as categorical variables.

CI, confidence interval; eGFR, estimated glomerular filtration rate; IS agents, immunosuppressive agents; RAAS, renin-angiotensin-aldosterone system.

Table S4. Microhematuria and Risk of Relapse in the SPHTCM Center

Factor	Hazard Ratio for Relapse (95% CI); *P* value			
	**Unadjusted**	**Model A**	**Model B**	**Model C**
**Initial hematuria** (per 1 unit greater)	1.25 (0.85-1.86); 0.262	1.27 (0.85-1.90); 0.234	1.51 (1.16-1.95); 0.002	1.57 (1.00-2.48); 0.053
**Initial persistent hematuria**	1.14 (0.44-2.93); 0.792	1.28 (0.48-3.39); 0.619	1.25 (0.45-3.49); 0.667	1.65 (0.56-4.90); 0.367

The initial hematuria, defined as the average hematuria of the first 6 months, was log-tranformed. In order to avoid data loss, 0.1 was added to the value of initial hematuria. The initial persistent hematuria, defined as a initial hematuria value of >5 RBCs/HPF, was expressed as a categorical variable. Model A was adjusted for age, sex, and hypertension; sex and hypertension were expressed as categorical variables. Model B was adjusted for covariates in Model A plus baseline serum albumin, eGFR, and proteinuria. Model C was adjusted for covariates in Model B plus use of RAAS blockades and IS agents. Use of RAAS blockades and IS agents were expressed as categorical variables.

CI, confidence interval; eGFR, estimated glomerular filtration rate; IS agents, immunosuppressive agents; RAAS, renin-angiotensin-aldosterone system.

Table S5. Worsening Hematuria and Risk of Short-Term Relapse in Cox Proportional Hazards Model C

Variable	Hazard Ratio	95% Confidence Interval	P Value
Appearance of positive hematuria	4.64	3.29-6.54	<0.001
Negative conversion of hematuria	0.67	0.53-0.84	0.001
Age	1.00	0.99-1.02	0.599
Male gender	1.22	0.78-1.91	0.374
Hypertension	0.83	0.53-1.29	0.404
Albumin	0.86	0.63-1.18	0.355
Proteinuria	1.04	0.98-1.12	0.218
EGFR	1.00	0.99-1.02	0.655
IS agents treatment			
Monotherapy	1.67	0.79-3.55	0.182
Combination therapy	1.18	0.59-2.36	0.642
RAAS blockade treatment	1.19	0.74-1.91	0.469

Appearance of positive hematuria and negative conversion of hematuria were treated as binary variables and included as time-varying covariates.

EGFR, estimated glomerular filtration rate; ESRD, end-stage renal disease; IS agents, immunosuppressive agents; RAAS, renin-angiotensin-aldosterone system.

Table S6. Microhematuria and Risk of Renal Progression in Competing Risk Regression Model C

Variable	Hazard Ratio	95% Confidence Interval	P Value
Time-averaged hematuria	1.35	1.12-1.63	0.002
Age	1.03	1.00-1.06	0.042
Male gender	0.95	0.47-1.92	0.882
Hypertension	0.94	0.52-1.71	0.841
Albumin	0.83	0.49-1.40	0.479
Time-averaged proteinuria	1.34	1.09-1.66	0.006
EGFR	0.99	0.98-1.01	0.214
IS agents treatment			
Monotherapy	2.13	0.69-6.59	0.189
Combination therapy	1.68	0.61-4.63	0.317
RAAS blockade treatment	1.07	0.56-2.06	0.828

Time-averaged hematuria and proteinuria were log-transformed due to their positively skewed distribution. To avoid data loss due to transformation, 0.1 was added to the values of time-averaged hematuria and proteinuria. Time-averaged hematuria and proteinuria were included as time-varying covariates.

EGFR, estimated glomerular filtration rate; ESRD, end-stage renal disease; IS agents, immunosuppressive agents; RAAS, renin-angiotensin-aldosterone system.

Table S7. Characteristics of Patients Grouped by Centers

Variable	XH	SPHTCM	P value
No. of patients	474	165	
**Baseline**			
Age (yr)	48 (34-57)	54 (45-64)	<0.001
Males, n (%)	305 (64.35)	104 (63.03)	0.096
BMI[a] (kg/m^2)	24.98 (22.32-27.38)	23.87 (21.97-25.73)	<0.001
Hypertension, n (%)	167 (35.23)	69 (41.82)	0.131
Nephrotic syndrome, n (%)	264 (55.70)	47 (28.48)	<0.001
Serum creatine, mg/dL	0.89±0.22	0.79±0.25	<0.001
eGFR (ml/min per 1.73 m^2)	94.75±20.41	99.27±20.59	0.015
Serum albumin (g/dL)	2.84±0.72	2.98±0.84	0.046
Microhematuria (RBCs/HPF)	3 (2-8)	4 (1-8)	0.840
Proteinuria (g/24 h)	3.67 (1.75-6.00)	2.51 (1.80-3.56)	<0.001
**Follow-up**			
Follow-up duration (mo)	41.5 (29-50)	36 (25-48)	0.005
Initial hematuria (RBCs/HPF)	3.19 (2-5.8)	3.5 (1.5-8)	0.034
TA-H (RBCs/HPF)	2.17 (1.14-3.47)	2.87 (1.84-4.30)	<0.001
CD-H (mo)	3 (1-7)	3 (1-11)	0.027
TA-P (g/24h)	1.06 (0.61-1.81)	1.69 (1.02-2.29)	<0.001
RAAS blockades, n (%)	378 (79.45)	118 (71.52)	0.029
IS agents			<0.001
Monotherapy, n (%)	48 (10.13)	45 (27.27)	
Combination therapy, n (%)	372 (78.48)	62 (37.58)	
**Outcome**			
No remission, n (%)	34 (7.17)	39 (23.64)	<0.001
Partial remission, n (%)	440 (92.83)	126 (76.36)	<0.001
Complete remission, n (%)	330 (75.00)	55 (43.65)	<0.001
Relapse, n (%)	90 (20.45)	21 (16.67)	0.345
Renal progression[a], n (%)	39 (8.23)	11 (6.67)	0.604
ESRD, n (%)	7 (1.48)	2 (1.21)	0.804

BMI, body mass index; CD-H, cumulative duration of hematuria; eGFR, estimated glomerular filtration rate; ESRD, end-stage renal disease; HPF, high power field; IS agents, immunosuppressive agents; RAAS, renin-angiotensin-aldosterone system; TA-H, time-averaged hematuria; TA-P, time-averaged proteinuria.

[a] Renal progression was defined as a 40% decline in eGFR or ESRD.

Table S8. Microhematuria and Risk of Renal Progression in the XH Center

**Factor**	**Subdistribution Hazard Ratio for Renal Progression (95% CI); *P* value**			
	**Unadjusted**	**Model A**	**Model B**	**Model C**
**Time-averaged hematuria** (per 1 unit greater)	1.60 (1.28-1.99); <0.001	1.67 (1.33-2.09); <0.001	1.58 (1.24-2.01); <0.001	1.55 (1.21-1.98); 0.001
**Cumulative duration of hematuria** (per 1 unit greater)	1.36 (1.15-1.61); <0.001	1.35 (1.17-1.56); <0.001	1.29 (1.10-1.50); 0.001	1.27 (1.09-1.48); 0.002

Renal progression was defined as a 40% decline in eGFR or ESRD. Death without renal progression was treated as a competing event. TA-H, CD-H, and TA-P, included as time-varying covariates, were log-tranformed. In order to avoid data loss, 0.1 was added to the values of TA-H and CD-H. Model A was adjusted for age, sex, and hypertension; sex and hypertension were expressed as categorical variables. Model B was adjusted for covariates in Model A plus baseline serum albumin, baseline eGFR, and TA-P. Model C was adjusted for covariates in Model B plus use of RAAS blockades and IS agents. Use of RAAS blockades and IS agents were expressed as categorical variables.

CD-H, cumulative duration of hematuria; CI, confidence interval; eGFR, estimated glomerular filtration rate; ESRD, end-stage renal disease; HPF, high power field; IS agents, immunosuppressive agents; RAAS, renin-angiotensin-aldosterone system; TA-H, time-averaged hematuria; TA-P, time-averaged proteinuria.

Table S9. Microhematuria and Risk of Renal Progression in the SPHTCM Center

Factor	Subdistribution Hazard Ratio for Renal Progression (95% CI); *P* value			
	**Unadjusted**	**Model A**	**Model B**	**Model C**
**Time-averaged hematuria**<br>(per 1 unit greater)	1.38 (1.15-1.66);<br>0.001	1.38 (1.13-1.69);<br>0.002	1.13 (0.84-1.53);<br>0.420	1.12 (0.82-1.54);<br>0.466
**Cumulative duration of hematuria**<br>(per 1 unit greater)	1.13 (0.98-1.32);<br>0.101	1.13 (0.97-1.32);<br>0.114	1.04 (0.90-1.20);<br>0.569	1.04 (0.90-1.20);<br>0.591

Renal progression was defined as a 40% decline in eGFR or ESRD. Death without renal progression was treated as a competing event. TA-H, CD-H, and TA-P, included as time-varying covariates, were log-tranformed. In order to avoid data loss, 0.1 was added to the values of TA-H and CD-H. Model A was adjusted for age, sex, and hypertension; sex and hypertension were expressed as categorical variables. Model B was adjusted for covariates in Model A plus baseline serum albumin, baseline eGFR, and TA-P. Model C was adjusted for covariates in Model B plus use of RAAS blockades and IS agents. Use of RAAS blockades and IS agents were expressed as categorical variables.

CD-H, cumulative duration of hematuria; CI, confidence interval; eGFR, estimated glomerular filtration rate; ESRD, end-stage renal disease; IS agents, immunosuppressive agents; RAAS, renin-angiotensin-aldosterone system; TA-H, time-averaged hematuria; TA-P, time-averaged proteinuria.

Table S10. Microhematuria and Risk of 50% Decline in Renal Fuction or ESRD

Factor	Subdistribution Hazard Ratio for Renal Progression (95% CI); *P* value			
	**Unadjusted**	**Model A**	**Model B**	**Model C**
**Time-averaged hematuria** (per 1 unit greater)	1.55 (1.25-1.91); <0.001	1.56 (1.25-1.95); <0.001	1.35 (1.09-1.66); 0.005	1.38 (1.12-1.71); 0.002
**Cumulative duration of hematuria** (per 1 unit greater)	1.35 (1.04-1.75); 0.026	1.30 (1.04-1.63); 0.019	1.23 (0.99-1.53); 0.062	1.23 (1.00-1.51); 0.055

Death without a 50% decline in eGFR or ESRD was treated as a competing event. TA-H, CD-H, and TA-P, included as time-varying covariates, were log-tranformed. In order to avoid data loss, 0.1 was added to the values of TA-H and CD-H. Model A was adjusted for age, sex, and hypertension; sex and hypertension were expressed as categorical variables. Model B was adjusted for covariates in Model A plus baseline serum albumin, baseline eGFR, and TA-P. Model C was adjusted for covariates in Model B plus use of RAAS blockades and IS agents. Use of RAAS blockades and IS agents were expressed as categorical variables.

CD-H, cumulative duration of hematuria; CI, confidence interval; eGFR, estimated glomerular filtration rate; ESRD, end-stage renal disease; IS agents, immunosuppressive agents; RAAS, renin-angiotensin-aldosterone system; TA-H, time-averaged hematuria; TA-P, time-averaged proteinuria.

Table S11. Characteristics of Patients Grouped by Hematuria Remission

Variable	Hematuria Remission	Hematuria Nonremission	P Value
No. of patients, n	71	149	
**Baseline**			
Age (yr)	48 (34-58)	50 (38-59)	0.614
Males, n (%)	46 (64.79)	107 (71.81)	0.290
BMI (kg/m^2)	24.00 (22.27-25.91)	24.83 (22.31-26.42)	0.416
Hypertension, n (%)	24 (33.80)	55 (36.91)	0.653
Nephrotic syndrome, n (%)	31 (43.66)	80 (53.69)	0.164
Serum creatine, mg/dL	0.84±0.21	0.88±0.24	0.299
EGFR (ml/min per 1.73 m^2)	100.12±19.69	96.24±21.40	0.198
Serum albumin (g/dL)	2.78±0.73	2.66±0.72	0.239
Microhematuria (RBCs/HPF)	8 (3-18)	5 (2-15)	0.204
Proteinuria (g/24 h)	2.86 (1.72-4.99)	3.55 (2.07-5.20)	0.313
**Follow-up**			
Follow-up duration (mo)	41 (27-52)	33 (23-46)	0.113
Initial hematuria (RBCs/HPF)	7.25 (3.6-10.28)	5.5 (3-10.97)	0.369
TA-H (RBCs/HPF)	3.87 (3.33-4.90)	5.19 (4.18-7.68)	<0.001
CD-H (mo)	8 (6-13)	11 (6-20)	0.026
TA-P (g/24h)	1.41 (0.88-2.71)	2.10 (1.15-3.01)	0.019
RAAS blockades, n (%)	57 (80.28)	101 (67.79)	0.054
IS agents			0.862
Monotherapy, n (%)	13 (18.31)	28 (18.79)	
Combination therapy, n (%)	46 (64.79)	100 (67.11)	
**Outcome**			
No remission, n (%)	13 (18.31)	48 (32.21)	0.031
Partial remission, n (%)	58 (81.69)	101 (67.79)	0.031
Complete remission, n (%)	35 (60.34)	56 (55.46)	0.548
Relapse, n (%)	24 (41.38)	54 (53.47)	0.142
Renal progression[a], n (%)	5 (7.04)	28 (18.79)	0.067
ESRD, n (%)	1 (1.41)	7 (4.70)	0.223

BMI, body mass index; CD-H, cumulative duration of hematuria; eGFR, estimated glomerular filtration rate; ESRD, end-stage renal disease; HPF, high power field; IS agents, immunosuppressive agents; RAAS, renin-angiotensin-aldosterone system; TA-H, time-averaged hematuria; TA-P, time-averaged proteinuria.

[a] Renal progression was defined as a 40% decline in eGFR or ESRD.

Table S12. Hematuria Remission and Risk of Renal Progression in Competing Risk Regression Model C

Variable	Hazard Ratio	95% Confidence Interval	P Value
Hematuria remission	0.63	0.41-0.96	0.034
Age	1.03	1.00-1.06	0.037
Male gender	0.63	0.25-1.61	0.335
Hypertension	1.50	0.74-3.02	0.257
Albumin	0.61	0.30-1.24	0.168
Time-averaged proteinuria	1.33	0.97-1.81	0.074
EGFR	0.99	0.97-1.01	0.538
IS agents treatment			
Monotherapy	4.94	0.75-32.63	0.098
Combination therapy	3.43	0.60-19.61	0.166
RAAS blockade treatment	0.96	0.42-2.18	0.920

Hematuria remission, included as a time-varing covariate, was the absence of hematuria or the presence of ≤3 RBCs/HPF in all the urine sediment tests performed during at least 12 months before the last outpatient visit. Time-averaged proteinuria was log-transformed and included as a time-varying covariate.

EGFR, estimated glomerular filtration rate; ESRD, end-stage renal disease; IS agents, immunosuppressive agents; RAAS, renin-angiotensin-aldosterone system.
